# Prevention in nursing care: a study protocol of a cluster-randomized controlled trial on the effects of physical exercise and environmental interventions on physical activity behavior and physical functioning in nursing home residents (PROGRESS study)

**DOI:** 10.3389/fragi.2025.1466315

**Published:** 2025-08-19

**Authors:** Vera Belkin, Tanja I. Janssen, Julian Rudisch, Bettina Wollesen, Claudia Voelcker-Rehage

**Affiliations:** ^1^ Department of Neuromotor Behavior and Exercise, Institute of Sport and Exercise Sciences, University of Münster, Münster, Germany; ^2^ Institute of Movement Therapy and Movement-Oriented Prevention and Rehabilitation, German Sports University Cologne, Cologne, Germany; ^3^ JICE, Joint Institute for Individualisation in a Changing Environment, University of Münster and Bielefeld University, Münster, Germany

**Keywords:** stationary care, physical activity, activities of daily living, cognition, well-being, health behavior, life space, activity tracker

## Abstract

**Background:**

Nursing home residents’ health and psychosocial well-being may be influenced by their often-sedentary lifestyles, which arise due to physical barriers like steps, an unwelcoming environment, limited awareness of the importance of physical activity (PA), and a lack of orientation. While exercise interventions are important for maintaining or improving physical and cognitive functions, they may not help increase daily PA behavior. Therefore, the PROGRESS study aims to investigate the short- and long-term effectiveness of tailored and combined physical exercise and environmental interventions for improving PA behavior and physical functioning among nursing home residents.

**Methods:**

The study employs a cluster-randomized controlled crossover design with four intervention groups. Participants will be randomly assigned to one of four groups: a combined exercise and guided environmental intervention (physical activity-promoting culture), an exercise intervention, a guided environmental intervention, or a non-guided environmental intervention. The exercise intervention involves group-based sessions twice a week, while the environmental intervention focuses on implementing PA into daily routines. The guided environmental intervention is equivalent to the non-guided one but provides additional support two to four times weekly. We aim to recruit at least 120 participants from six nursing homes. Each facility will receive two interventions in a 16-week crossover design (36 weeks in total, including weeks for measurements), followed by a 16-week non-guided environmental intervention (follow-up phase). Primary outcomes include steps per day as an indicator of PA behavior, and the Short Physical Performance Battery (SPPB) to assess physical functioning.

**Discussion:**

We hypothesize that the physical activity-promoting culture will yield superior effects than either intervention alone on the primary outcomes. Furthermore, both the guided environmental intervention and the exercise intervention are expected to be more effective than the non-guided environmental intervention. We aim to encourage nursing home residents to actively participate in social life and to derive guidelines for health promotion.

**Trial registration:**

The study is registered at the German Clinical Trial Register (DRKS) under registration number de DRKS00031020 (23.02.2023) and has received ethical approval from the University of Münster, Faculty of Psychology and Sports Science (2022-40-CVR).

## Introduction

The aging process is associated with a decline in physical and cognitive abilities as well as a higher incidence of adverse health events. Therefore, the older population tends to have more problems with activities of daily living (ADL) and impaired psychosocial well-being (Guralnik et al., 1993). Demographic changes mean that the average age of the population is increasing, leading to a greater number of highly aged individuals who will require support and care. The number of people in Germany who require care is currently around 4.1 million, and this care is provided for both outpatient (home care) and stationary (nursing home) settings (de Jong et al., 2023). Due to physical and cognitive impairments, most nursing home residents require assistance in their daily lives, and some even experience severe loss of independence, necessitating intensive care (Stockel et al., 2017; Bischoff et al., 2023). Therefore, it is crucial to promote health among older adults who need care, particularly those with multiple health conditions (Araujo de Carvalho et al., 2017).

The decline in physical and cognitive abilities and the increase of adverse health events tend to happen alongside sedentary behavior and a less active lifestyle (Provencher et al., 2008), therewith inducing a vicious cycle. Sedentary behavior reduces physical and cognitive functioning not only due to less cognitive and physical stimulation but also because of fewer social interactions (Schrempft et al., 2019; Giurgiu et al., 2020). Age-related cognitive decline affects almost all dimensions of cognition, including spatial cognitive abilities like spatial orientation and navigation (Moffat et al., 2001). This, in turn, can hinder an active lifestyle, especially one’s mobility within a nursing home and its environment (life space mobility, LSM).

Physical and cognitive abilities as well as psychosocial well-being can be maintained or improved even in older adults suffering from multiple health problems by appropriate interventions such as physical exercise (Mulrow et al., 1994; Kryger and Andersen, 2007; Pedersen and Saltin, 2015; Cordes et al., 2021a). However, the type, design, and duration of the training must be considered to achieve training effects. For example, interventions that combine cognitive and physical exercise components tend to more strongly benefit both motor and cognitive performance than interventions focused on pure motor or cognitive training (Wollesen and Voelcker-Rehage, 2014). Combined physical and cognitive training interventions have been shown to improve physical abilities, including physical strength, cardiorespiratory fitness, flexibility, balance, and everyday functionality and mobility (Fiatarone et al., 1990; Sherrington et al., 2008; Liu and Latham, 2009; Marmeleira et al., 2009; Gine-Garriga et al., 2010; Matsuda et al., 2010; Steib et al., 2010). Furthermore, these interventions have been demonstrated to reduce falls and physical frailty. They also seem to benefit cognitive functions (Marmeleira et al., 2009), including visual attention, executive functioning, and speed of information processing. With respect to the design, duration, and frequency, intervention studies that adapt to individuals’ abilities, focus on personal resources and are conducted twice a week for 45–60 min have been found to have the most positive effects (Wollesen and Voelcker-Rehage, 2014; Cordes et al., 2021a).

Beyond exercise interventions, promoting daily physical activity (PA) in nursing homes seems to be an urgent issue, as nursing home residents do not get sufficient PA in their normal lives. For example, Auerswald et al. (2022) investigated the PA behavior of nursing home residents using activity trackers (Fitbit Zip). On average, nursing home residents took 1.020 steps per week (–600 m) and spent 9 h per day sitting or lying down. At least two and a half of these hours were spent sitting or lying down continuously (see (Clark and Sugiyama, 2015; Vallance et al., 2018) for similar results). These findings indicate that the daily lives of nursing home residents lack PA, which can harm their health. Research has shown that there is a correlation between the amount of time spent sitting and the risk of mortality (Ebeling et al., 2013; Ministry of Health, 2013).

Only a few approaches have aimed to improve daily PA behavior. Integrated into a classic group training program, Wollesen et al. developed a training concept for care facilities in which both physical skills and spatial orientation are practiced (Wollesen et al., 2020a). Programs developed within long-term care institutions have demonstrated that individualized exercise regimens can be both safe and effective, even when integrated into routine care practices (Braun et al., 2015; Cordes et al., 2021b), virtual cycling systems (D'Cunha et al., 2021), and virtual reality training (Scheicher et al., 2017) to encourage movement and increase engagement. Second, physical space modifications include dynamic lighting (Baandrup and Jennum, 2021) or enriched gardens (Bourdon and Belmin, 2021). Thirdly, group activity spaces used giant exercise board games (Mouton et al., 2017), outdoor exercise parks with age-friendly stations (Levinger et al., 2023), or indoor exercise stations (Fleiner et al., 2015; van Delft et al., 2020). Specifically, Fleiner and colleagues (2015) set up an “active corridor” in different wards of a geriatric psychiatric clinic, with posters instructing patients on how to exercise. Patients were asked to walk in the active corridor for 20 min, four times a day, three times a week. In a different study, Van Delfts’s group used a step-by-step method (Grol and Wensing, 2015) to develop an “action plan” for four wards of a hospital in Uetrecht. The plan was developed and implemented with the involvement of patients, physiotherapists, nurses, doctors, and patients’ relatives (van Delft et al., 2020). This included the creation of exercise guides and material, as well as 7-min workout videos for lying, sitting, and standing, and QR-code walking routes throughout the building. The reported performance measures showed improvements in physical activity as well as physical function in most cases. For example, group activity spaces recorded gains in mobility, balance, and strength (Scheicher et al., 2017; Buckinx et al., 2020; Bourdon and Belmin, 2021; Mak et al., 2022). Incorporating such environmental interventions within a nursing home could increase PA behavior and promote social contact, as well as improve spatial orientation and LSM.

So far, studies have focused on either group-based physical exercise interventions or environmental interventions. While both intervention models show positive results, exercise interventions primarily promote physical functioning, whereas environmental interventions positively affect physical activity. Therefore, we suggest combining group-based physical exercise interventions with approaches to explicitly increase PA levels. As such, we developed this protocol to offer a new intervention model, namely, a physical activity-promoting culture, which combines both types of interventions: group-based physical exercise classes and environmental interventions. We hypothesize that the physical activity-promoting culture might lead to larger effects on PA behavior and physical functioning in nursing home residents than each intervention alone. Additionally, we expect that, individually, the guided environmental intervention and the exercise intervention will be more effective than the non-guided environmental intervention. Thus, the overall aim of the PROGRESS study is to encourage active participation in social life among nursing home residents and to develop guidelines for health promotion.

## Methods

The SPIRIT statement was used as a guideline for this protocol paper (Chan et al., 2013) (see Supplementary Table S1).

### Trial design

**FIGURE 1:**
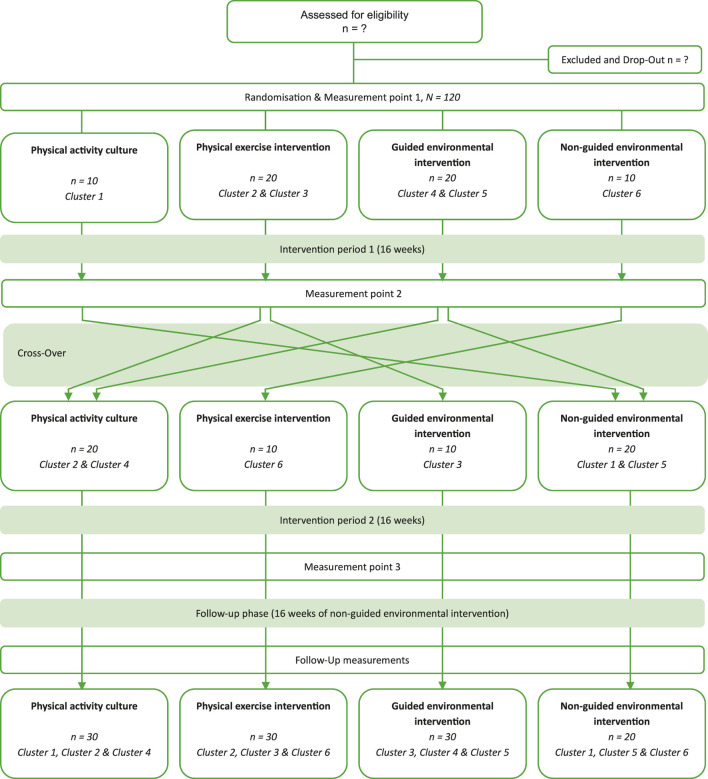
Study design- CONSORT Flow-Diagram of the PROGRESS project. Note: The power calculation determined that a total of 116 participants were required. To improve traceability, this number has been rounded up to 120.

Based on existing research and the physical exercise recommendations mentioned above, this monocentric intervention study is conducted through a controlled cluster-randomized crossover design with four arms. The four interventions are randomly paired and ordered using MATLAB (‘nchoosek’ and ‘randperm’ functions), ensuring that each intervention will be combined with each of the other three interventions once. These six different intervention combinations were then clustered randomized to the nursing homes in the agreed order. In general, the trial design can be divided into an intervention and a follow-up phase with four separate weeks of measurements (t1-t4). So t1 is the baseline measurement time point, t2, with a double role, corresponds to the post-measurement point for the first intervention as well as the pre-measurement point for the second intervention phase. According to this, t3 will be the post-measurement point for the second intervention. 16 weeks after t3, the study will be concluded with the follow-up measurements (t4) after 52 weeks (see Figure 1). The date of first enrollment was August 2022.

### Participants

#### Ethics approval and trial registration

The study is being conducted according to the Declaration of Helsinki and the guidelines of Good Clinical Practice (GCP). All participants will give written informed consent before enrollment and are allowed to leave the study at any time without any consequences. The ethics committee of the Faculty of Psychology and Sports Science of the University of Münster, Germany, has approved the study protocol (2022-40-CVR). The study was registered at DRKS. de with registration number DRKS00031020 on 23.02.2023.

#### Recruitment of nursing homes

The study has aimed to identify at least six nursing homes around Münster, Germany, that are willing to take part. To ensure enough participants, institutions were selected based on their number of residents (ideally >100), as we aimed to recruit 10–20 participants per nursing home (n = 116) and 29 per intervention arm (see power calculation). The first point of contact was with the nursing home management via email or telephone. During this initial contact, the possibility of participating in a study to promote physical activity was enquired about, without providing any details. Participation is voluntary and not remunerated. Nursing home recruitment ended in the third quarter of 2023.

#### Recruitment of participants

Once a nursing home expresses interest in participating, a standardized information event will be held to introduce the PROGRESS project to all interested residents, staff, and relatives of residents. The information event will also serve as a needs assessment, as initial ideas for promoting a more active lifestyle will be collected. After the event, a nursing home employee will be asked to list all residents who meet the inclusion criteria and are interested in participating. Each nursing home resident on the list will be informed about the project and the conditions of participation in a standardized one-on-one meeting conducted by a research assistant. This meeting will follow the structure of the agreement paper, and it will be checked whether the participants are cognitively able to follow the conversation. Any outstanding issues will be clarified, and if the participant fits the eligibility criteria, consent to participate will be obtained. If a nursing home resident is unable to sign documents, the consent meeting will be conducted with the legal guardian. Participants will be blinded; they will not be aware of interventions in other nursing homes, and they will not be in contact with residents from other nursing homes.

#### Eligibility criteria

Inclusion criteria are: (i) voluntary participation, (ii) ability to participate in group training, (iii) minimum age of 60 years, (iv) ability to understand and implement instructions given by a trainer, and (v) ability to sit freely in a (wheel)chair. No other criteria are applied. If a study participant no longer fulfills the inclusion criteria due to a natural, age-related deterioration in general condition, they will be excluded from study participation (but they will be welcome to continue participating in the interventions voluntarily).

#### Assignment of interventions

Assessments will be performed by blinded investigators in a pseudonymized manner to ensure blind data collection and analysis. To avoid performance bias, measurements and interventions will follow a standardized protocol. The participating nursing homes (clusters) will be randomly assigned to the interventions by the project staff in the agreed order. The sports scientist or physiotherapist delivering the intervention will only receive the names of the participants in the intervention group without being aware of the design.

#### Outcome measures

The assessments focus on two key domains (primary outcomes) to evaluate the efficacy of the interventions (see Table 1): physical activitiy and physical functioning. Further assessments (secondary outcomes) evaluate physical and subjective performance measures, activities of daily living, cognition, psychological wellbeing and emotions, intention to be physically active, and stage of change. All measurements are listed in the SPIRIT diagram in Table 1.

**TABLE 1:** Spirit diagram - Schedule of activities.

			Study period					
			Enrollment	Allocation	Baseline	Posttests		Follow-up
TIMEPOINT			*-t*_*1*_	*t*_*0*_	*t*_*1*_	*t*_*2*_	*t*_*3*_	*t*_*4*_
Enrollment	**Eligibility screen**		X					
	**Informed consent**		X					
	**Allocation**			X				
Intervention	Physical activity culture				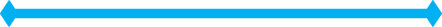			
	Physical exercise intervention				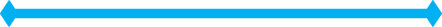			
	Guided environmental intervention				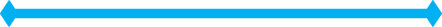			
	Non-guided environmental intervention				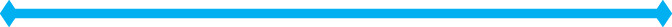			
Assessments	*Primary Outcomes*							
	PA	Steps per day			X	X	X	X
	PF	SPPB			X	X	X	X
	*Secondary Outcomes*							
	PP	Hand grip strength			X	X	X	X
		Frailty			X	X	X	X
		Gait patterns			X	X	X	X
		Posture			X	X	X	X
	ADL	Barthel Index			X	X	X	X
		NNA			X	X	X	
		NHLSD			X	X	X	X
	COG	MoCA			X	X	X	
		Auditory Task			X	X	X	X
		Gait patterns DT			X	X	X	X
		Posture DT			X	X	X	X
		LMR			X	X	X	X
	HB	Subjective age			X	X	X	
		Subjective health			X	X	X	X
		Short-FES-I			X	X	X	
		PSC scale			X	X	X	X
	PWB	DIA-S			X	X	X	
		CES-D			X	X	X	
		EQ-5D-3L			X	X	X	
		WHOQOL			X	X	X	X
	Int	Intention			X	X	X	

Note: X implementation of the assessment; the line with two dots represents the period of the intervention phase with starting and endpoint, t-1 before pretest, t1 pretest, t2 and t3 posttests, and t4 follow-up. I1, Intervention 1; I2, Intervention 2; PA, Physical activity; PF, Physical functioning; PP, Physical performance measures, Frailty Fried Index; ADL, Activities of daily living; COG, Cognition; HB, Health behavior; PWB, Psychological well-being and emotions; Int, Intention; SPPB, Short Physical Performance Battery; NAA, Nuernberg-Ageing-Everyday-Life Test; NHLSD, Nursing Home Life Space Diameter; MoCA, Montreal Cognitive Assessment; LMR, Landmark Recognition Task; PSC scale, Psychological Self-Concept Scale; DIA-S, Depression in Age Scale; CES-D, Center for Epidemiological Studies Depression Scale; EQ-5D-3L, European Quality of Life; WHOQOL, World Health Organization’s Quality of Life, Intention Intention to be physically active and stage of change.

#### Screening

*Demographics.* Demographic characteristics will be assessed to describe the sample, including age (years), body height (m), body mass (kg), and body mass index (BMI). Additionally, we will screen for sex, leg length (relevant for the GAITRite gait analysis system), education, objective health (diseases and medications), COVID-19 infections, and use of assistive aids.

*Number of falls.* The nursing staff will have documented the number of falls occurring within the last 4 months.

### Primary outcomes

#### Physical activity (PA)

The *Fitbit Zip* (Fitbit, San Francisco, United States) device will be used to objectively track PA, and its 6-month battery life supports prolonged use. The Fitbit Zip has been validated for use in different age groups and conditions (Tully et al., 2014; Sharp et al., 2017; Streber et al., 2017), and it has been shown to deliver data that is considerably more accurate than subjective measures and close to that of the “gold standard” ActiGraph (Meyer et al., 2019). The Fitbit will be attached to participants’ clothes and will record their steps taken and distance traveled, which can be viewed on the Fitbit’s display. Participants will be instructed to wear the Fitbit Zip as often as possible throughout the project duration (52 weeks), immediately after waking up, and to remove it before going to bed. The data will be synced weekly; malfunctioning devices will be replaced (Tully et al., 2014; Sharp et al., 2017; Streber et al., 2017). To analyze steps per day, hours with at least one step will be identified as wearing hours (Meyer et al., 2016). Days with less than 6 h (identified as less than 6 h with at least one step) or not fulfilling the “three-a-day” criterion (using the tracker at least once in the morning, once around noon, and once in the afternoon, identified as at least one step having been registered) will be excluded from PA calculations. These days will be classified as non-wearing days, according to the heuristic of Meyer and colleagues (Meyer et al., 2017). Sedentary minutes will be defined as periods of at least 20 min with three or fewer steps per minute (Meyer et al., 2019). To calculate sedentary time and the *longest zero* time, we will exclude data from days with less than 6 hours of wearing. To express sedentary minutes as a percentage of total waking time, waking time will be defined as a 12-h period (Alessi et al., 2005). This definition of waking time is necessary because it is difficult to distinguish between non-wearing time and sedentary time (Meyer et al., 2017). Usage behavior will be quantified as the proportion of days worn.

#### Physical functioning

The Short Physical Performance Battery (SPPB) (Guralnik et al., 1994) assesses lower-extremity function via balance, gait speed, and sit-to-stand performance. Balances will be tested across three progressively challenging stances (parallel (0–1 point), semi-tandem (0–1 point), and tandem stand (0–2 points); balance sum 0–4 points). To get into the specified position, participants will be allowed to hold on to an aid; however, aids are not allowed during the measurement. Gait speed will be recorded over 4 m at a comfortable pace, with an aid being used if necessary (0–4 points). Leg strength will be measured by the time taken to complete five chair rises (0–4 points). Individual scores will be summed up to a total from 0 (low mobility/functionality) to 12 points (full mobility/functionality). A change of ∼1.0–1.3 points is considered clinically meaningful (Guralnik et al., 1994).

### Secondary outcomes

#### Objective performance measures

*Hand grip strength.* Hand grip strength will be measured using a digital hydraulic hand dynamometer (Grip-D T.K.K. 5101 by Takei, Japan) with three trials per hand. Participants will hold the device in a standardized seated position (device placed on the subject’s lap, elbow bent between 100–110°, and the hand dynamometer gripped from above) for each attempt. After confirming handedness, three attempts per hand will be recorded; we will evaluate both peak value as well as the average value of the dominant hand, for the frailty scoring (Fried et al., 2001).

*Frailty* follows Fried’s five-criterion phenotype (2001), predicting adverse outcomes. They comprise (i) unintentional weight loss of more than 4.5 kg within the last year, (ii) self-reported fatigue (CES-D), and (iii) low physical activity (via Fitbit). Low activity is defined per Fried’s sex-specific kcals/week thresholds (men <383 kcal/week; women <270 kcal/week). Additional criteria are (iv) slow walking speed (<4.57 m in 7 s) and (v) weak hand grip strength (sex-specific cut-points; men <5.85 kg; women <3.37 kg). According to Fried’s Cardiovascular Health Study criteria, 0 points indicates robustness (no frailty), 1-2 points indicates pre-frailty, and 3-5 points indicates frailty (Fried et al., 2001; Braun et al., 2016).

*Gait patterns*. Gait will be assessed by measuring the time needed to finish a 8.5-m track; we will assess step length, step width, gait speed, and double support phase using the portable gait analysis system GAITRite (GAITRite; CIR Systems Inc., Clifton, NJ, United States). Each participant will complete one trial. The data will be analyzed using the GAITRite System software (GAITRite; CIR Systems Inc., Clifton, NJ, United States, Version 4.8.5) to calculate the average values of each parameter for each measurement.

*Posture.* Participants’ posture will be examined using a force plate (AMTI Force and Motion, Model: BP400600HF-2000, Serial Number: 5239, Advanced Mechanical Technology Inc., Watertown, MA, United States). Participants will be asked to stand as still as possible in a predefined position for 15 s (Donath et al., 2016). During the measurement, we will record fluctuations in the center of pressure (COP) in the medio-lateral and anterior-posterior directions.

#### Activities of daily living

*Activities of daily living (reported by others).* The Barthel Index evaluates basic activities of daily living (ADL) independence. Ten items will yield a 0–100 score reflecting dependency (Mahoney and Barthel, 1965). The items of the Barthel Index will be completed by the nursing staff.

*Activities of daily living (self-report).* The Nuernberg-Ageing-Everyday-Life Test *(NAA)* assesses daily living tasks. Seventeen NAA items relevant to nursing-home ADLs will be used (Oswald and Fleischmann, 1999). The items are scored by 1 “often” to 3 “never”. The total score ranges between 17–51 points; ≤32 points (63.3%) denotes independence.

*Life Space mobility*. The Nursing Home Life-Space Diameter (NHLSD; Tinietti and Ginter, 1990) quantifies residents’ movement across four facility zones. It tracks mobility changes and intervention effects, and it has shown acceptable inter- and intra-rater reliability. The four zones are: the resident’s private room (zone 1), the ward in which they reside (zone 2), the rest of the facility (zone 3), and the area outside the facility (zone 4). Zone-visit frequencies 0 (never) to 5 (more than three times a day) are weighted by zone level and doubled for unassisted movement (Tinetti and Ginter, 1990).

#### Cognition

*Global cognition.* The Montreal Cognitive Assessment (MoCA) is a brief 30-point screening tool for mild cognitive impairment and early stages of Alzheimer’s disease. It evaluates key domains including memory, executive function, attention, language, and orientation (Wong et al., 2018). Scores ≤25 indicate possible mild impairment; higher scores are considered normal (Nasreddine et al., 2005).

*Cognitive performance while performing single and dual tasks*. Cognitive performance will be assessed via an auditory task in which residents judge six landmarks as ‘inside’ or ‘outside’ the facility. Pre-standardized audio files (selection procedure via MATLAB R2022a, recording using the Audacity^®^ program version 3.4.1) will present the landmark in randomized order for 15–18 s. Responses (‘yes’/‘no’) will be recorded across three randomized landmark distributions to avoid bias (3 inside: 3 outside, 2 inside: 4 outside, and 1 inside: 5 outside). An initial practice will ensure audibility and comprehension. The task will be performed alone and concurrently with walking (8.5 m of walking) and posture tasks (15 s of standing still); correct responses and motor metrics will be analyzed as mentioned above.

*Spatial orientation.* Spatial orientation will be measured via a 12-item landmark recognition (LMR) test (DeIpolyi et al., 2007; Wollesen et al., 2020a). Participants must identify the four genuine facility landmarks among 12 images representing all life space zones (Jansen et al., 2017). Additionally, four “fake” images will depict similar locations elsewhere. The order of the 12 pictures will be randomly generated using MATLAB (MATLAB R2022a). Scores will be reflected in correct identifications (+1 point), unsure (0 points), and false alarms (−1 point). Total scores range from −12 to 12 points; higher scores indicate better orientation.

#### Health behavior

*Subjective age*. Participants’ subjectively perceived age will be compared to their actual current age to assess their image of age and their perceived age norms. In response to the question “How old do you feel?“, residents will answer with their perceived age.

*Subjective health*. Participants’ health behavior will be assessed with one item from the Health Survey SF12 (Ware et al., 1998). Participants will be asked to rate their general health on a 5-point Likert scale ranging from excellent to poor.

*Concerns about falls*. The Short-FES-I (Hauer et al., 2011) measures fall-related concern across daily tasks. Three non-applicable items will be omitted (items 1, 3, and 13). Each remaining question will be rated from 1 = “not at all concerned” to 4 = “very concerned.”

*Physical self-concept*. To capture residents’ physical self-concept we will use an adapted scale of the Physical Self-Concept Scale (PSC scale) by Stiller et al. (2004). We will focus on three two-item subscales (endurance, strength, and coordination), rated 1 (“disagree”) to 4 (“agree”). Mean scores will be computed for each subscale.

#### Psychological well-being and emotions

*Depression.* The Depression in Age Scale (DIA-S) screens depression in geriatric patients (Heidenblut and Zank, 2010). 10 yes/no items will assess depressive symptoms over the past 14 days. If statements 1–2, 4-6, and 8-9 are answered affirmatively and statements 3, 7, and 10 are answered negatively, all 10 depressive symptoms are confirmed. Scores ≥4 indicate probable depression; scores ≤3 are inconclusive.

*Loneliness*. Loneliness is assessed by a single Center for Epidemiological Studies Depression Scale (CES-D) item (Radloff, 1977). Residents will be asked to answer the question “Have you felt lonely in the last week?” with one of the four possible answers: “rarely or not at all (<1 day)”, “sometimes (1–2 days)”, “often (3–4 days)”, “most of the time to all of the time (5–7 days).”

*Health-related quality of life.* The European Quality of Life 5 Dimensions 3 Level Version (EQ-5D-3L) assesses health-related quality of life across five dimensions: mobility, self-care, usual activities, pain/discomfort, and anxiety/depression. Levels range from 1 (‘no problems’) to 3 (‘extreme problems’) per dimension (Brooks, 1996). A five-digit profile (e.g., 12,211) will be generated and converted into a utility score. EQ-5D-3L profiles will be converted to utility values using the German values of the European Value Study (EVS) (Greiner et al., 2005). Utilities range from 1.00 (11,111; perfect health state) to −0.36 (33,333; worst health state).

*Quality of life.* The World Health Organization’s Quality of Life (WHOQOL) assessment defines quality of life as individual’s self-related life satisfaction (WHO, 1998). A single item (“How would you describe your quality of life?”) will be rated on a 1 (‘very bad’) to 5 (‘very good’) scale.

#### Intention to be physically active and stage of change

Participants’ intention to engage in physical activity PA and their subjective status of physical activity will be assessed via two questions. Intention will be rated on a 1 to 7 agreement scale. Stage of activity (≥2.5 h/week) will be classified into five intention/adoption categories (Lippke et al., 2009).

## Interventions

We are following a participatory approach to developing and conducting environmental interventions in nursing homes. Therefore, 1 month before t1 in each participating nursing home, a workshop was conducted with representatives from all status groups. The goal was to develop a participatory strategy to integrate more PA-supporting structures into the residents’ living spaces. During the workshop, the life space mobility model by Jansen and colleagues was explained (Jansen et al., 2017). Based on that model, we determined the actual status and the desired status for the most frequently visited places in the nursing home. The results were compared to identify potential mismatches. Workshop members were then asked to identify resources, wishes, and problems to eliminate barriers and stimulate motivation for residents to leave zones 1 and 2 more often and be more physically active. Based on the workshop results, the research team developed suggestions for PA-promoting changes in the nursing home and its environment. These suggestions were reviewed by the nursing homes’ stakeholders, and suggestions for changes were incorporated before implementing the interventions (Conger and Kanungo, 1988; Cornwall and Jewkes, 1995; Jansen et al., 2017). All interventions follow the same general framework and aim to increase physical activity and physical functioning. Across all nursing homes and their residents, the interventions are tailored to the specific needs and spatial structures. This participatory process is supported by all stakeholders and adapted to the individual needs of the facilities.

A follow-up workshop will be held at each nursing home around t3 to evaluate the effectiveness of the interventions and discuss the implementation of sustainable PA promotion. An overview about all interventions is given in Table 2.

**TABLE 2:** Description of the four interventions.

Intervention	Components	Week 1–4	Week 5–8	Week 9–12	Week 12–16
**Physical exercise intervention (I1, I2)**	Mobilization and warm-up (5–10 min.)	Week 1–16 • Range of motion exercise for the wrists, hips, shoulders, wrists, knees, and ankles			
	Coordination, balance, and cognitive exercises (10 min.)	Week 1–16 (with weekly progression) • Standing balance, bodyweight shifting • Simple cognitive tasks • Coordinative exercises with small equipment and everyday materials (e.g., balls, scarves) • Spatial orientation exercises, e.g., pointing to cardinal directions, assigning rooms of the facility to a map • Orientation puzzle			
	Gait training (18–20 min.)	•75–90 m • Direction pointing task	•105–120 m • Direction pointing task	•135–150 m • Direction pointing task	•165–180 m • Direction pointing task
	Muscle strengthening exercises (10 min.)	• Chair rises • Upper body and trunk exercises with additional materials and weights • Functional lower-limb exercises	cf. Week 1–4 • Individual progression of number of repetitions		
	Cool down (5–10 min.)	Week 1–16 • Stretching • Relaxation exercises			
**Guided environmental intervention (I1, I3)**	Indoor (Life-space 2–3)	Week 1–16 • Trim paths through the facility • Movement posters focusing on mobilization, strengthening, and balance • Seated bicycles and other exercise equipment • Information brochure on trim path • Before implementation: 20-minute guided training session for nursing staff • Guided group activities offered 2/week by a project worker and opportunity to be performed independently			
	Outdoor (Life-space 4)	-	-	Week 9–16 • Booklet with 4 outdoor routes (500 m to 1,500 m) • Barrier-free • Written descriptions, photos, and map • Should be walked independently • Guided group walks offered 2/week	
**Non-guided environmental intervention (I4)**	Indoor (Life-space 2–3)	Week 1–16 • Trim paths through the facility • Movement posters focusing on mobilization, strengthening, and balance • Seated bicycles and other exercise equipment • Information brochure on trim path • Before implementation: 20-minute guided training session for nursing staff • Should be performed independently			
	Outdoor (Life-space 4)	-	-	Week 9–16 • Booklet with 4 outdoor routes (500 m to 1,500 m) • Barrier-free • Written descriptions, photos, and map • Should be walked independently	

Note: I1 Intervention 1, Physical activity culture (combination of physical exercise intervention and guided environmental intervention); I2 Intervention 2, Physical exercise intervention; I3 Intervention 3, Guided Environmental intervention; I4 Intervention 4, Non-guided environmental intervention.

### Combined physical exercise and guided environmental intervention (physical activity-promoting culture)

During the physical activity-promoting culture intervention, exercise and guided environmental interventions as described below will be synergistically implemented in a participatory process. Additional information can be found in Table 2.

### Physical exercise intervention

The exercise program intervention will consist of 32 sessions of 45–60 min each. The intervention will be conducted over 16 weeks and offered twice per week. Group sizes will be up to 10 participants per training session. One certified exercise scientist or physiotherapist will conduct every session and be supported by an assistant. The programme was developed following the guidelines of the International Association of Gerontology and Geriatrics (IAGG), and the exercise intervention is based on the *PROfit orientation* program by Wollesen et al. (2020b). This programme aims to improve PA, LSM, and spatial orientation. The program will be modified for an intervention period of 16 weeks (formerly 12 weeks) and adapted to the nursing home settings. Further, the program will be continuously adapted to residents’ abilities and needs. Exercises that do not work well will be replaced by more appropriate and acceptable exercises, while the target of the replaced exercises should remain the same. To increase motivation and participation, incentives such as stamp cards and monthly awards certificates for the most frequent participation will be introduced. Further details are provided in Table 2.

### Guided environmental intervention

The environmental intervention will focus on material-structural environmental changes, as well as creating supply and availability structures for PA (Franzkowiask, 2022). The overall aim is to improve PA both inside and outside the facility. Therefore, engaging and stimulating exercise stations will be created at different locations in the care facility that can be independently used by residents. These stations will be developed in a participatory manner and designed under consideration of exercise principles. Examples of exercise stations are exercise posters or exercise stations with equipment like balls or walking routes. Following the principle of nudging, these posters/exercise stations will be placed at multiple locations in the nursing home so that residents are invited to be active in different life spaces of the nursing home (Eichhorn, 2019). Further, they might feel invited to expand their LSM by visiting exercise stations in life space zones 2–3 (Jansen et al., 2017). As a further motivational factor, exercise stations can be integrated into paths with different levels of difficulty (just zone 2 up to zones 2 + 3). To integrate zone 4 into the environmental intervention, a brochure containing a map, text, and pictures of different outdoor routes will be developed to help residents find their way around the area outside the nursing home. The intervention will run for 16 weeks, and it will be promoted by a sports scientist two to four times a week for 45–60 min each session. The sports scientist will explain the exercise stations to residents, supervise their use, and provide guidance on correcting motor movements, offering motivation, and adapting exercises according to the residents’ needs and abilities. More information on the guided environmental intervention is presented in Table 2.

### Non-guided environmental intervention

The non-guided environmental intervention will use the same materials as the guided environmental interventions. However, this intervention will not be supervised or guided, so participants must engage independently, with the assistance of nursing home employees or relatives. This non-guided environmental intervention also serves as the follow-up condition between t3 and t4 (see Table 2).

## Data management and analysis

### Data management

Electronic data will be stored on a university server that is backed up on a hard drive using the SPSS software. Only the leading project staff will have access to the server. Data that is originally in paper format and has been digitized will be stored separately in a locked cabinet. The data will be stored and identified only by pseudonymized ID codes. The code list with the participants’ names and combined ID codes will only be available to the leading project staff and will be stored separately from the data in a locked cabinet. Double-checking and plausibility checks will be performed to verify data quality.

### Statistical analysis

The descriptive statistics will be presented as either group mean values and standard deviations/errors or medians and interquartile ranges, depending on the distribution of the outcome measures. The intervention effect for the primary outcomes (Number of Steps and SPPB) will be analyzed separately using a 4 (between group: PA-promoting culture intervention, guided environmental intervention, non-guided environmental intervention, or exercise intervention) × 2 (within test time point: t1:t2, and t2:t3) linear mixed model, where participants can be nested within nursing homes and be calculated as a random effect. The Follow-Up effects will be analyzed additionally using a 4 (between group: all interventions) x 3 (within test timepoint: t1/t2 – t2/t3 – t4). If baseline differences are found, the baseline assessment values, as well as age and sex discrepancies, will be included as covariates in the statistical model. In cases of statistically significant interaction effects, Bonferroni-adjusted *post hoc* tests (such as t-tests or Wilcoxon tests) will be employed to identify significant differences between groups from baseline to post-allocation testing. The data will be pre-processed and analysed using SPSS.

The effect size will be determined using Cohen’s f, which indicates the magnitude of treatment effectiveness and helps assess whether a statistically significant difference is practically significant (Cohen and Ebl, 1988). Cohen’s f values are classified as small (0 ≤ f ≤ 0.24), medium (0.25 ≤ f ≤ 0.39), or large (f ≥ 0.40) (Grissom and Kim, 2011). For *post hoc* analysis, Cohen’s d effect size will be calculated. Additionally, PSdep scores (probability of superiority for dependent samples) will be calculated as an estimate of effect size in non-parametric *post hoc* tests. The significance level will be set at p < 0.05. An intention-to-treat analysis will be conducted, considering all participants in the groups to which they were randomly assigned, regardless of whether they received or adhered to the assigned intervention. We will control participation in the interventions to analyze the effect of the intervention in more detail. Multiple imputations will be used to handle missing data, assuming that the missingness is random. If more than 40% of participants are lost to outcome assessment or demonstrate insufficient participation in training sessions, an additional per-protocol analysis will be conducted.

### Sample size estimate/power calculations

Statistical power analysis was conducted to estimate the sample size to test our hypothesis in G*Power (Version 3.1.9.4, Heinrich Heine University of Duesseldorf) (Faul et al., 2009). The following input parameters were used to obtain small-sized test × group interaction effects within an ANOVA: effect size (*f* = 0.15) (i.e., a small effect, (Tudor-Locke et al., 2011), type I error (α = 0.025), type II error (1-β = 0.8), number of groups (n = 4), number of measurements (n = 2), correlation between measurements (r = 0.60).

The size of the estimated sample to generate sufficient power to achieve the small effect is N = 88. To compensate for the estimated dropout rate of 30% (e.g., 20% lost to follow-up; plus 10% mortality) we plan to include a total of N = 116 participants. Based on the study design, we will integrate N = 29 participants in each condition.

Regarding the special cohort, recruiting approximately 30 participants per intervention arm per nursing home seems unrealistic, so multiple nursing homes will be needed for each intervention arm. As the number of nursing homes is also limited, each one needs to take on more than one intervention arm to reach a total of ∼30 participants. By the life expectancy of the special cohort and for ethical reasons, we have limited the maximum study duration to 1 year per participant. This means that each participant can complete two intervention phases and one follow-up phase per year, nested in a nursing home. In terms of the four intervention arms, this means six different possible combinations (this means six different nursing homes), each with 10–20 nursing home residents participating in the study.

### Data monitoring

For the trial, no data monitoring committee is needed because all interventions are conducted by skilled and trained instructors, and no instructor has an interest in the specific outcomes of any intervention. Furthermore, the leading project staff will observe the participants. If any sign of indisposition, pain, dizziness, or states of confusion appear during measurements at any point, the project staff will intervene and stop the procedure or intervention. If any participant has a significant event (e.g., a fall) that could affect their participation in the study, the nursing home staff will inform the project staff and offer documentation and interpretation of the results.

## Discussion

This cluster-randomized trial aims to assess the impact of a physical activity-promoting culture, compared to three other interventions, on nursing home residents’ PA behavior and physical functioning. We expect the physical activity-promoting culture, which combines exercise classes and environmental changes, to increase PA, reduce sedentary behavior, and improve physical functioning (Giurgiu et al., 2020; Lopponen et al., 2021) more effectively than either intervention alone or a non-guided environmental intervention. Overall, the PROGRESS study offers a new practical approach to implementing active aging in nursing homes. It aims to encourage the integration of PA programs led by professionally educated instructors into the prevention planning of nursing homes, together with environmental changes that might promote PA behavior in general. The participatory approach enables residents, nursing staff, social services, and relatives to be aware of and incorporate any changes in the residents’ environment. This approach is hypothesized to promote physical functioning and activity, even in challenging situations. A physical activity-promoting culture that is lively, happy, and healthy can only be developed if all individuals who work, live, and visit the nursing home support each other through PA. The findings can provide guidance for addressing current and future challenges in health promotion initiatives in long-term care, which is of great importance in aging societies.

When residents are mobile, able to take care of themselves, and challenged and encouraged in their environment, they might become more physically active (den Ouden et al., 2017). Staying active is believed by many older adults to enrich their overall life experiences (Guell et al., 2016; Halaweh et al., 2018), and data from Sococco et al. shows that only 5.9% of residents move into a nursing home based on their own decision (Scocco et al., 2006). However, the decrease in obligations as well as physical and cognitive limitations that often come along with moving into a nursing home can lead to immobility and dependence on others, as well as an increase in sedentary behavior. As seniors become sedentary and less physically active, their physical and cognitive abilities often decline rapidly (Auerswald et al., 2020). Due to spending most of their time in one place, typically their room (Jansen et al., 2017), their spatial orientation abilities also decrease (Wollesen et al., 2020a), leading to isolation, loneliness, and depression (Antoniewicz and Brand, 2016) and a decline in quality of life (Saajanaho et al., 2016; Duppen et al., 2020). Most studies that have examined the effects of physical exercise on these health outcomes (Fiatarone et al., 1990; Crocker et al., 2013; Johnen and Schott, 2018; Cordes et al., 2019) have revealed that multicomponent training can affect physical and cognitive outcomes, such as walking, balance, dual-tasking abilities, and wellbeing (Wollesen and Voelcker-Rehage, 2014). Furthermore, maintaining physical functioning related to ADLs can promote independence (Saajanaho et al., 2015). This, in turn, could relieve the burden on nurses and reduce the gap in demand between care and nurses, especially given the current nursing shortage. Exercise interventions in nursing homes are typically conducted twice a week for about 1 hour each session (Cordes et al., 2021a). However, engaging in PA only twice a week for about 45–60 min may not promote residents’ daily PA behaviors and their LSM, and it may not reduce the time they spend sitting in one place: Nursing home residents spend most of their time sitting regardless of whether they participate in group sessions once or twice a week (Auerswald et al., 2020). Thus, the unique contribution of PROGRESS is to combine an exercise intervention with an environmental intervention. We assume that both making changes in the nursing home environment and having residents participate in exercise classes will contribute to residents’ physical functioning, PA, and mobility (Broers et al., 2017; Blaga et al., 2018; Kwan et al., 2020; Hernandez et al., 2023). While the exercise intervention aims to have participants practice certain physical and cognitive functions based on well-known exercise principles, such as intensity and progression, the environmental intervention is particularly aimed at removing obstacles in the environment that residents face. Since this intervention equips the entire nursing home with exercise stations, PROGRESS may reduce nursing home residents’ sedentary behavior and improve their LSM. Further, both types of interventions aim to help residents establish new social contacts. Also, because motivational factors are important determinants of exercise participation and PA, we follow a participatory approach to make sure participants are interested in engaging in these behaviors, overcoming motivation barriers. An additional unique aspect of PROGRESS is its follow-up phase, which will last for 16 weeks after the intervention is completed (after t3, week 37–53). This phase aims to evaluate the program’s sustainability and related changes in primary and secondary outcomes. (Fiatarone et al., 1990; Crocker et al., 2013; Johnen and Schott, 2018; Cordes et al., 2019). It is expected that during the follow-up period, the primary and secondary outcomes will worsen but not return to the baseline level.

This study has several limitations that should be acknowledged. First, while Fitbit trackers were used to objectively assess physical activity, periods with zero recorded steps may not exclusively indicate sedentary behavior but could also reflect times when the device was not worn. Moreover, brief interruptions or incomplete wear periods may have influenced the calculation of total wear days, potentially leading to an underestimation of actual activity levels. Second, the absence of a traditional control group limits the ability to attribute observed effects solely to the interventions, as natural fluctuations in physical activity or contextual factors cannot be ruled out. Third, adherence to the non-guided environmental intervention cannot be systematically controlled. Furthermore, in the case of the guided environmental intervention, only attendance at the supervised sessions can be monitored, but not when the participants practice independently. Nevertheless, we could interview the nursing staff to assess whether these interventions lead to the anticipated increase in mobility. In contrast, the other interventions are supervised, and the use of new devices is guided – an important aspect, as guidance has been shown to be a key factor for PA behavior (Antoniewicz and Brand, 2016).

## Data Availability

The raw data supporting the conclusions of this article will be made available by the authors, without undue reservation.
